# Inhibition of NLRP3 Inflammasome Activation and Pyroptosis in Macrophages by Taraxasterol Is Associated With Its Regulation on mTOR Signaling

**DOI:** 10.3389/fimmu.2021.632606

**Published:** 2021-02-17

**Authors:** Fan Yang, Xun-jia Ye, Ming-ye Chen, Hong-chun Li, Yao-feng Wang, Mei-yan Zhong, Chun-su Zhong, Bo Zeng, Li-hui Xu, Xian-hui He, Dong-yun Ouyang

**Affiliations:** ^1^Department of Immunobiology, College of Life Science and Technology, Jinan University, Guangzhou, China; ^2^Wuzhongpei Memorial Hospital of Shunde, Foshan, China; ^3^Department of Cell Biology, College of Life Science and Technology, Jinan University, Guangzhou, China

**Keywords:** taraxasterol, dandelion, NLRP3 inflammasome, ASC speck, mTOR

## Abstract

Taraxasterol (TAS) is an active ingredient of Dandelion (*Taraxacum mongolicum* Hand. -Mazz.), a medicinal plant that has long been used in China for treatment of inflammatory disorders. But the underlying mechanism for its therapeutic effects on inflammatory disorders is not completely clear. Inflammasome activation is a critical step of innate immune response to infection and aseptic inflammation. Among the various types of inflammasome sensors that has been reported, NLR family pyrin domain containing 3 (NLRP3) is implicated in various inflammatory diseases and therefore has been most extensively studied. In this study, we aimed to explore whether TAS could influence NLPR3 inflammasome activation in macrophages. The results showed that TAS dose-dependently suppressed the activation of caspase-1 in lipopolysaccharide (LPS)-primed murine primary macrophages upon nigericin treatment, resulting in reduced mature interleukin-1β (IL-1β) release and gasdermin D (GSDMD) cleavage. TAS greatly reduced ASC speck formation upon the stimulation of nigericin or extracellular ATP. Consistent with reduced cleavage of GSDMD, nigericin-induced pyroptosis was alleviated by TAS. Interestingly, TAS time-dependently suppressed the mechanistic target of rapamycin (mTOR) complex 1 (mTORC1) and mTORC2 signaling induced by LPS priming. Like TAS, both INK-128 (inhibiting both mTORC1 and mTORC2) and rapamycin (inhibiting mTORC1 only) also inhibited NLRP3 inflammasome activation, though their effects on mTOR signaling were different. Moreover, TAS treatment alleviated mitochondrial damage by nigericin and improved mouse survival from bacterial infection, accompanied by reduced IL-1β levels *in vivo*. Collectively, by inhibiting the NLRP3 inflammasome activation, TAS displayed anti-inflammatory effects likely through regulation of the mTOR signaling in macrophages, highlighting a potential action mechanism for the anti-inflammatory activity of Dandelion in treating inflammation-related disorders, which warrants further clinical investigation.

## Introduction

One critical response of the innate immune system against pathogenic infections and aseptic damages is the activation of various inflammasomes, which are induced by pathogen-associated molecular patterns (PAMPs) and/or damage-associated molecular patterns (DAMPs) (1). What sense the PAMPs or DAMPs are pattern recognition receptors (PRRs) on the cell membrane, or within the cytosol (including organelle membranes). Many of the PRRs can transduce the signals of PAMPs or DAMPs into the cytosol and nucleus to induce inflammatory response-related gene expression, but some of them, such as nucleotide binding oligomerization domain (NOD)-like receptors (NLRs), rather than inducing gene expression, can instead be triggered to form inflammasome as a platform for caspase-1 auto-catalytic activation, resulting in not only the maturation and release of pro-interleukin-1β (IL-1β) and pro-IL-18, but also a lytic cell death named pyroptosis, both of which induce robust inflammatory responses to confer protection against infections (2). Thus, the formation and activation of inflammasome is an important first line of defense against pathogenic infections and aseptic tissue injury of the host.

Among the known inflammasomes, the NLR family pyrin domain containing 3 (NLRP3) inflammasome is the most extensively explored one (3). During NLRP3 inflammasome activation, LPS priming increases the expression of pro-IL-1β and NLRP3, while a second signal provided by DAMPs such as extracellular ATP and nigericin can fully activated the inflammasome. Under the stimulation of second signal, a constitutively-expressed protein ASC (apoptosis-associated speck-like protein) is recruited by NLRP3 to form a large complex named ASC speck, which is a key step for pro-caspase-1 activation and a hallmark of inflammasome activation, leading to the cleavage of pro-IL-1β and gasdermin D (GSDMD). Pyroptosis, an inflammatory form of cell death, is induced due to the membrane perforating property of the GSDMD N-terminal fragment (GSDMD-NT) derived from GSDMD by caspase-1 cleavage. NLRP3 inflammasome activation thus results in a robust inflammatory response and plays important roles in defending against bacterial, fungal and viral infections. On the contrary, dysregulation of NLRP3 inflammasome activation has been implicated in various inflammatory diseases, such as neurologic disorders (multiple sclerosis, Alzheimer's disease, and Parkinson's disease) and metabolic diseases (type 2 diabetes, obesity, gouty arthritis and atherosclerosis), in addition to infectious diseases (4). Thus, NLRP3 is a potential target for therapeutic intervention of these related disorders. For example, by targeting directly to the NATCH domain of NLRP3, MCC950 has been shown to effectively block NLRP3 inflammasome assembly and attenuate the severity of experimental autoimmune encephalomyelitis in a mouse model of multiple sclerosis (5). Other NLRP3 pathway-regulating molecules including endogenous small molecules (6, 7) and chemicals (8–12) or natural products (13–17) have also been identified to inhibit NLRP3 inflammasome activation *in vitro* in macrophages and *in vivo* in animal models.

As NLRP3 inflammasome activation represents a strong inflammatory response, it is tightly regulated at many steps of its activation by various signaling pathways including protein kinase A (PKA), c-Jun N-terminal kinases (JNKs) and mechanistic target of rapamycin (mTOR) signaling (18, 19). Some of the NLRP3 inhibitors (such as MCC950 and oridonin) have been shown to block the assembly of NLRP3 inflammasome by directly targeting NLRP3 protein and ion flux, while others including phytochemicals from medicinal plants have been revealed to suppress NLRP3 inflammasome assembly by modulating the post-translational modification (such as phosphorylation, ubiquitination and succinylation) of NLRP3 inflammasome components or inducing signaling pathways including PKA (6, 7, 16, 17). Previous reports have indicated that NLRP3 inflammasome inducers trigger vigorous immunometabolic (mTOR signaling and glycolysis) changes in macrophages, which constitutes a new avenue to regulate NLRP3 inflammasome activation (20). Supporting this, mTOR inhibitors such as rapamycin and Torin 1 have been identified to suppress NLRP3 inflammasome activation (20, 21). Considering the critical role of NLRP3 inflammasome activation, identification of new NLRP3 inflammasome inhibitors is still intriguing to develop drugs or prototypes for treatment of NLRP3-related disorders.

Taraxasterol (TAS) is an active ingredient of Dandelion (*Taraxacum mongolicum* Hand.-Mazz.), a medicinal plant that has long been used in oriental medicine for treatment of inflammatory diseases. Various anti-inflammatory activities of TAS have been reported, including anti-tumor (22), protection from hepatic (23) and neural injuries (24), anti-infection (25), anti-endotoxic shock (26), anti-rheumatoid arthritis (27) and other inflammatory diseases (28–30). As the above-mentioned inflammatory disorders have been implicated with either bacterial infections or aseptic inflammations (25, 27), it is likely that NLRP3 inflammasome may have been involved in. Therefore, we hypothesized that TAS might exhibit anti-inflammatory effects by influencing the activation of NLRP3 inflammasome, although other mechanisms including upregulating Hint1 expression (22), inhibiting NF-κB signaling and reducing inflammatory cytokine secretion (23, 30) have already been reported. Our results showed that TAS inhibited the NLRP3 inflammasome activation by suppressing the assembly of ASC specks in murine macrophages, leading to reduced release of IL-1β and decreased pyroptotic cell death. Such inhibitory effect on the assembly of NLRP3 inflammasome by TAS was probably mediated through the mTOR signaling.

## Materials and Methods

### Reagents

TAS was purchased from Yuan-Ye Bio-Technology (Shanghai, China), dissolved in absolute ethyl alcohol at 5 mM just before use. Opti-MEM, Dulbecco's Modifed Eagle's Medium (DMEM) medium with high glucose, fetal bovine serum (FBS), Lipofectamine 2000 (11668-030), penicillin and streptomycin were purchased from ThermoFisher/Gibco (Carlsbad, CA, USA). Rapamycin (R8781), dimethyl sulfoxide (DMSO) (D8418), propidium iodide (PI) (P4170), Hoechst 33342 (B2261), adenosine triphosphate (ATP) (A6419), and LPS (from *Escherichia coli* O111:B4) (L4391) were purchased from Sigma-Aldrich (St. Louis, MO, USA). Nigericin, Pam3CSK4, Poly(dA:dT) and flagellin (FLA-PA ultrapure) were obtained from InvivoGen (San Diego, CA, USA). FuGENE HD transfection reagent (E2311) was bought from Promega (Madison, WI, USA). INK-128 was obtained from Active Biochemicals (Hong Kong, China). Antibodies against pro-caspase1+p10+p12 (ab179515), GSDMD (ab209845) were bought from Abcam (Cambridge, UK). The anti-NLRP3 antibody (AG-20B-0014) was purchased from Adipogen AG (Liestal, Switzerland). Antibodies against IL-1β (#12242), ASC (#67824), phospho-Akt (Ser473) (#4060), phospho-Akt (Thr308) (#2965), Anti-Akt (pan) (#2920), p70S6K1 (#2708), phospho-p70S6K1 (#9234), phospho-4E-BP1 (#2855), 4E-BP1 (#9644), horse-radish peroxidase (HRP)-linked horse anti-mouse IgG (#7076) and HRP-linked goat anti-rabbit IgG (#7074) were products of Cell Signaling Technology (Danvers, MA, USA). The anti-actin antibody (sc-1616-R) was purchased from Santa Cruz Biotechnology (Dallas, TX, USA). CF568-conjugated goat-anti-rabbit IgG (H+L), highly cross-adsorbed (#20103) were obtained from Biotium (Hayward, CA, USA).

### Animals

Female C57BL/6 mice (6–8 weeks of age) were purchased from the Experimental Animal Center of Southern Medical University (Guangzhou, China). All the mice were acclimatized for 1 week before experiment. The animal study was reviewed and approved by the Committee on the Ethics of Animal Experiments of Jinan University.

### Cell Line Culture

Mouse macrophage line J774A.1 was obtained from the Kunming Cell Bank of Type Culture Collection, Chinese Academy of Sciences (Kunming, China). Cells were cultured in complete DMEM medium (DMEM plus 10% FBS and 1% penicillin/streptomycin) at 37°C in a humidified incubator of 5% CO_2_.

### Bone Marrow-Derived Macrophages (BMDMs) Differentiation

The separation and differentiation of mouse BMDMs was performed as previously reported (31, 32). In brief, mice were sacrificed by cervical dislocation and sterilized by 70% ethanol. The bone marrow cells in hind femora and tibias were flushed out by 10 ml sterile PBS and separated from the mixture by centrifugation at 300 × g for 5 min at 4°C. Then the bone marrow cells were re-suspended by BM-Mac medium which consisted of 80% complete DMEM medium and 20% M-CSF-conditioned medium from L929 cells and cultured in the 10-cm petri dish with 10 ml BM-Mac medium at 37°C in a humidified incubator of 5% CO_2_. The bone marrow cells differentiated into BMDMs after 6 days. Then BMDMs were collected by using cell-scraper and cultured in 6-well plates at 1.2 × 10^6^ cells/well (2 ml) or 24-well plates at 1.5 × 10^5^ cells/well (0.5 ml) with complete DMEM medium at 37°C in a humidified incubator of 5% CO_2_. The cells were ready for experiments overnight.

### Thioglycolate-Elicited Peritoneal Macrophages Isolation

Thioglycolate (TG) (3%) was intraperitoneally injected into mice which were sacrificed by cervical dislocation 4 days later. The TG-elicited peritoneal macrophages (TGPMs) were isolated by washing buffer (sterile PBS plus 5% newborn calf serum and 0.5 mM EDTA). The cells were then separated from the mixture by centrifugation at 300 × g for 5 min and cultured in 6-well plates or in 24-well plates with complete DMEM medium. Two hours later, the medium containing suspended cells were discarded and attached cells (TG-elicited peritoneal macrophages) were cultured in fresh complete medium.

### Cell Death Assay

Cell death was measured by PI incorporation as previously described (33, 34). Briefly, the cells were cultured in 24-well plates overnight and replaced the complete medium for the Opti-MEM with LPS (0.5 μg/ml). Four hours later, the cells were treated with TAS for 1 h followed by stimulation with nigericin (2 μM for TGPMs, 3 μM for BMDMs) for 1 h. Then the PI (2 μg/ml) and Hoechst 33342 (5 μg/ml) mixture were added into the cells medium to stain the dead cells and the nuclei, respectively. The stained cells were observed by live imaging using Zeiss Axio Observer D1 microscope equipped with a Zeiss LD Plan-Neofluar 20×/0.4 Korr M27 objective lens (Carl Zeiss MicroImaging GmbH, Göttingen, Germany). Fluorescence images were captured with a Zeiss AxioCam MR R3 cooled CCD camera controlled with ZEN software (Carl Zeiss).

### Western Blot Analysis

Western blotting was performed as previously described (32). Briefly, whole cell lysates were separated by sodium dodecyl sulfate-polyacrylamide gel electrophoresis (SDS-PAGE) and electro-transferred to PVDF membranes (03010040001; Roche Diagnostics GmbH, Mannheim, Germany), which were blocked by blocking buffer for 1 h once the electro-transfer had been completed. Then, the membrane was incubated with primary antibody at 4°C overnight, followed by incubation with appropriate HRP-conjugated secondary antibody. The target bands were visualized with an enhanced chemiluminescence kit (BeyoECL Plus; Beyotime, Shanghai, China) and captured on X-Ray films. The results were recorded by FluorChem8000 imaging system (AlphaInnotech, San Leandro, CA, USA) and analyzed by AlphaEaseFC 4.0 software (AlphaInnotech).

### Precipitation of Soluble Proteins in Supernatants

The soluble proteins in the culture supernatants were precipitated as previously described (35). Then the precipitated pellets were washed thrice by cold acetone, and re-dissolved in 2× SDS-PAGE loading buffer. The target proteins were analyzed by Western blotting as above.

### Detection of Soluble IL-1β

Soluble IL-1β secreted into culture supernatants were detected by cytometric bead array (CBA) mouse IL-1β Flex Set (#560232; BD Biosciences, San Jose, CA, USA), with experimental methods being in line with the manufacturer's instructions. Data were acquired and analyzed by CELLQuest software on a flow cytometer (Attune NxT acoustic focusing cytometer, Thermo Fisher Scientific; Waltham, MA, USA).

### JC-1 Assay

BMDMs were primed with 0.5 μg/ml LPS for 4 h, Then the cells were pre-treated with or without TAS (100 μM) for 1 h, followed by stimulation with nigericin (5 μM) for 1 h. Mitochondrial membrane potential was evaluated with a JC-1 assay kit (Beyotime, Shanghai, China) according to the instructions of the manufacturer.

### Immunofluorescence Microscopy

Immunofluorescence analysis was performed as previously described (36). In brief, BMDMs were seeded in 24-well plates at 37°C. The next day, cells were primed with 0.5 μg/ml LPS for 4 h. Later, the cells were pre-treated with or without TAS, followed by stimulation with ATP (2 mM) for 30 min or nigericin (3 μM) for 1 h. Later, the cells were fixed with 4% paraformaldehyde for 15 min and permeabilized with 2 ml cold methanol at −20°C for 10 min. After permeabilization, the cells were blocked by blocking buffer, followed by incubation with anti-ASC antibodies overnight and staining with CF568-conjugated goat-anti-rabbit IgG for 1 h. Finally, the nuclei were stained by 5 μg/ml Hoechst 33342 solution for 5 min. The images were acquired through a Zeiss Axio Observer D1 microscope with a Zeiss LD Plan-Neofluar 40×/0.6 Korr M27 objective (Carl Zeiss) with Zeiss AxioCam MR R3 cooled CCD camera controlled with ZEN software (Carl Zeiss).

### Bacterial Infection

The bacterial infection animal model was established as previously reported (37). *E. coli* DH5α was prepared as previously described (38). Mice were randomly divided into 4 groups (*n* = 10 for mouse survival experiment; *n* = 5 for detection of cytokines). Briefly, mice were administered intragastrically (*i.g*.) with TAS (20 mg/kg body weight/mouse) or vehicle (2% Tween-80 in PBS); 3 h later, the mice were injected with freshly-prepared viable *E. coli* (2 × 10^9^ CFU/mouse in 0.3 ml PBS) into the peritoneal cavity of each mouse; 1 h after bacterial infection, the mice were given with a same dose of TAS again. Animal survival was observed and recorded every 6 h for 3 consecutive days. In a parallel experiment, mice were treated with TAS and infected with *E. coli* similarly; at 8 h post bacterial infection, the mice were sacrificed and the levels of IL-1β in the sera and peritoneal lavage fluids were evaluated by with a cytometric beads assay kit (BD Biosciences, San Jose, CA, USA) according to the instructions of the provider.

### Statistical Analysis

All data were represented as mean ± standard deviation (SD). To assess the statistical significance among multiple groups and between two groups, one-way analysis of variance (ANOVA) followed by Tukey *post-hoc* test and unpaired Student's *t*-test was executed using Graph Pad Prism software, respectively. In addition, Friedman or Mann-Whitney U was used to analyze the data that did not conform to the normal distribution. *P*-values < 0.05 were considered statistically significant.

## Results

### TAS Suppresses NLRP3 Inflammasome Activation in Murine Macrophages

**Figure 1:**
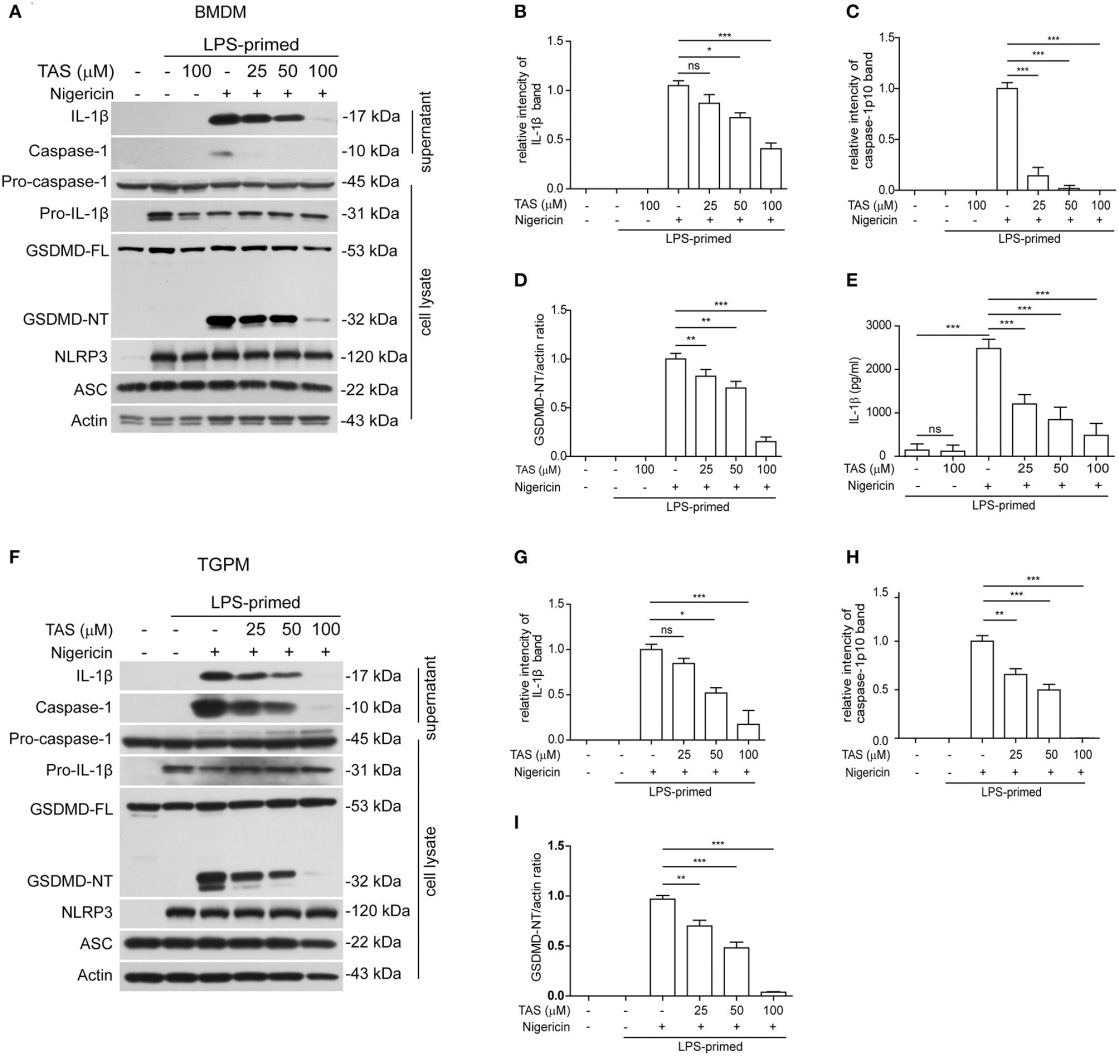
Taraxasterol (TAS) dose-dependently suppressed nigericin-induced activation of NLRP3 inflammasome in murine macrophages. Bone marrow-derived macrophages (BMDMs) **(A–E)** or thioglycollate (TG)-elicited peritoneal macrophages (TGPMs) **(F–I)** were primed with LPS (0.5 μg/ml) for 4 h and then pretreated with indicated concentrations of taraxasterol (TAS) for 1 h, followed by stimulation with nigericin (3 μM for BMDMs and 2 μM for TGPMs) for 1 h. **(A,F)** Western blot analysis of the expression levels of indicated proteins in the culture supernatants and cell lysates. **(B,C,G,H)** Histograms show the relative intensity of capase-1p10 (10 kDa) or mature IL-1β (17 kDa) bands in supernatants, with the intensity of nigericin group being set to 1.0, respectively. **(D,I)** Relative GSDMD_NT levels in the cell lysates. **(E)** The concentration of soluble IL-1β in the culture supernatants were detected by cytometric bead array (CBA) assay. Data were analyzed using the non-parametric Mann–Whitney U-test, which are shown as mean ± SD (*n* = 3). *P < 0.05; **P < 0.01; ***P < 0.001; ns, not significant.

To explore the influence of TAS on NLRP3 inflammasome activation, we pre-treated with or without TAS and then activated the inflammasome in LPS-primed BMDMs (Figures 1A–E) or TGPMs (Figures 1F–I) with the canonical NLRP3 activator nigericin. As expected, Western blot analysis indicated that the levels of pro-IL-1β and NLRP3 were significantly increased after LPS priming, while those of pro-caspase-1 and ASC were unaffected (Figures 1A,F). After nigericin stimulation, both caspase-1p10 (10 kDa) and mature IL-1β (17 kDa, a cleaved product by active caspase-1) were present in the culture supernatants of these macrophages, indicative of the activation of NLRP3 inflammasome. Interestingly, TAS pre-treatment for 1 h dose-dependently inhibited the release of mature IL-1β and caspase-1p10 from BMDMs (Figures 1A–C) or TGPMs (Figures 1F–H) stimulated with nigericin, while it did not induce the release of these proteins without nigericin stimulation (Figures 1A,F). Consistent with the Western blot analysis, the results of cytometric beads array (CBA) also showed that TAS suppressed nigericin-induced release of IL-1β from BMDMs (Figure 1E). Supporting the decrease of nigericin-induced caspase-1 activity, GSDMD cleavage (generation of GSDMD-NT) was also inhibited by TAS treatment (Figures 1A,D,F,I). Together, these results indicated that TAS inhibited NLRP3 inflammasome activation in LPS-primed murine macrophages upon nigericin treatment.

### TAS Blocks ASC Speck Formation in BMDMs Upon NLRP3 Inflammasome Stimulation

**Figure 2:**
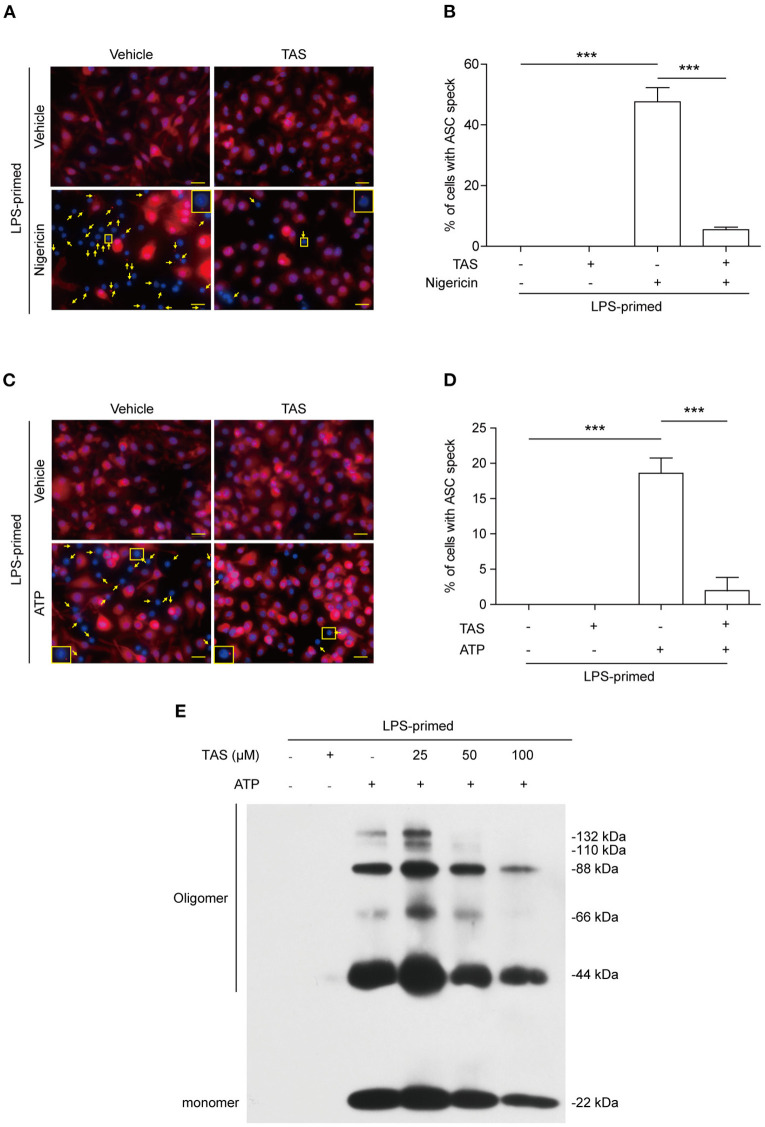
TAS inhibited ASC speck formation in macrophages. BMDMs were primed with LPS (0.5 μg/ml) for 4 h and then pretreated with TAS (100 μM) for 1 h, followed by stimulation with nigericin (3 μM) for 1 h **(A,B)** or ATP (2 mM) for 30 min **(C,D)**. **(A,C)** Representative immunofluorescence images showing ASC (red) subcellular distribution. Nuclei were stained with Hoechst 33342 (blue). The images for ASC and nuclei were captured, respectively, and merged together. Yellow arrows indicate ASC specks and the insets show the cell with an ASC speck. **(B,D)** Histograms show the percentage of cells with an ASC speck relative to total cells from 5 randomly chosen fields. Data are shown as mean ± SD (*n* = 5). ***P < 0.001; ns, not significant; scale bars, 20 μm. ASC oligomerization after ATP stimulation was assayed by Western blotting **(E)**.

Published studies have shown that the adaptor protein ASC can oligomerize into a large speck in each cell during the assembly of NLRP3 inflammasome (39). The formation of ASC specks can therefore be considered as another biomarker of NLRP3 inflammasome activation (40). We next explored whether TAS influenced ASC speck formation in BMDMs by using immunofluorescence microscopy analysis. The result showed that ASC was diffusely distributed in unstimulated cells while forming one speck in each cell upon nigericin stimulation, and that TAS markedly suppressed the ASC speck formation (Figure 2A). Quantitative analysis indicated that ASC specks were formed in ~48% of the LPS-primed BMDMs upon nigericin stimulation, but in the presence of TAS, only ~5% of the total cells were observed with a ASC speck (Figure 2B), corroborating that TAS inhibited NLRP3 inflammasome activation in nigericin-treated macrophages.

Apart from nigericin, extracellular ATP is another commonly-used activator for inducing canonical NLRP3 inflammasome assembly (41). Both nigericin and ATP trigger NLRP3 Inflammasome activation by inducing K^+^ efflux (42). But unlike nigericin which serves as a potassium ionophore directly mediates K^+^ efflux to trigger NLRP3 inflammasome activation, ATP acts as a ligand of plasma membrane P2X7 purinergic receptor to induce K^+^ efflux via the TWIK2 potassium channel (43). Immunofluorescence microscopy showed that ATP treatment induced the formation of ASC specks in LPS-primed macrophages (Figure 2C), but TAS markedly reduced the number of cells with ASC specks induced by ATP stimulation (Figures 2C,D). This phenomenon was corroborated by its effect on reducing the levels of ASC oligomers (Figure 2E). These results together suggested that TAS suppressed NLRP3 inflammasome activation by blocking the formation of ASC specks.

### TAS Suppresses Nigericin-Induced Pyroptosis in Macrophages

**Figure 3:**
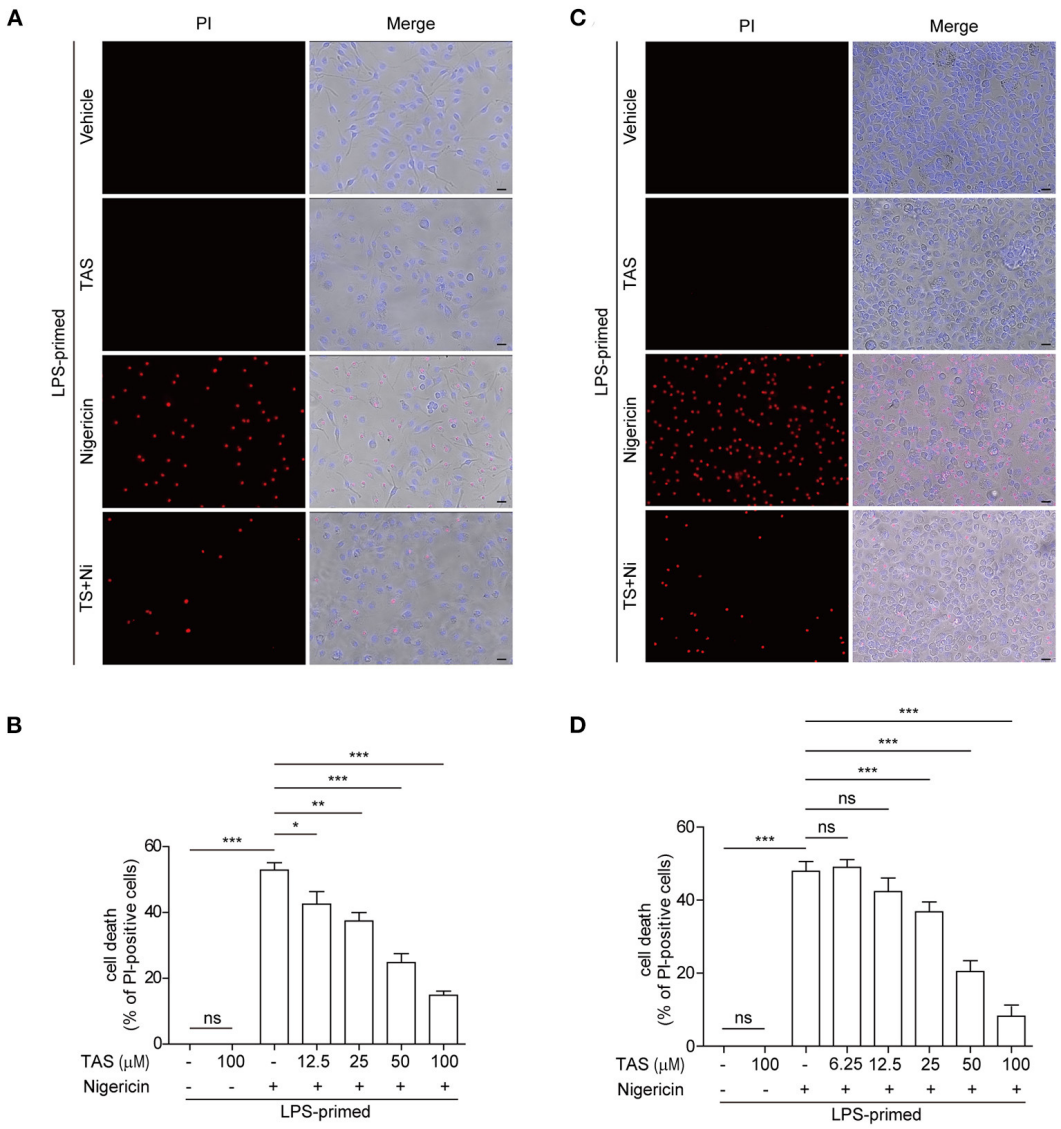
TAS dose-dependently suppressed nigericin-induced pyroptosis in macrophages. BMDMs **(A,B)** were treated as in Figure 1A and TGPMs **(C,D)** were treated as in Figure 1E. **(A,C)** After TAS and nigericin (Ni) treatment, BMDMs and TGPMs were stained with 2 μg/ml propidium iodide (PI) (red; staining dead cells) and 5 μg/ml Hoechst 33342 (blue; staining all cells) for 10 min, and then observed by fluorescent microscopy. Bright-field images are also shown in merged ones (here images of TAS = 100 μM are shown). **(B,D)** The percentage of cell death is defined as PI-positive cells to all cells (Hoechst 33342-positive) in 5 random fields. Data are shown as mean ± SD (*n* = 5). *P < 0.05; **P < 0.01; ***P < 0.001; ns, not significant; scale bars, 20 μm.

Apart from cleaving pro-IL-1β, activated caspase-1 can also cleave GSDMD to produce a GSDMD-NT fragment which can bind to and form pores in the plasma membrane, leading to a rapid lytic cell death named pyroptosis (44). The relative level of GSDMD-NT therefore represents a surrogate marker of caspase-1 activity and pyroptosis. As TAS had been shown to suppress nigericin-induced activation of caspase-1 (as revealed by caspase-1p10 release into the culture supernatants in Figure 1) upon NLRP3 inflammasome activation, we next explored whether TAS could also inhibit pyroptosis. Concomitant with the blockade of GSDMD-NT production (Figure 1), nigericin-induced lytic cell death, as revealed by propidium iodide (PI) incorporation, was dose-dependently decreased by TAS treatment in both BMDMs (Figures 3A,B) and TGPMs (Figures 3C,D), indicating that TAS was able to suppress NLRP3-mediated pyroptosis. TAS (100 μM) alone did not induce GSDMD-NT production (Figure 1) or lytic cell death in LPS-primed BMDMs or TGPMs (Figure 3), suggesting that the inhibitory effect of TAS on GSDMD-NT production upon NLRP3 inflammasome activation was not due to its cytotoxicity.

Besides NLRP3 protein, other inflammasome sensors including NLRC4 and AIM2 also exist in murine macrophages. We thus detected whether TAS influenced the activation of NLRC4 and AIM2 inflammasomes. Murine macrophage J774A.1 cells were first primed with TLR2/1 ligand Pam3CSK4, and then transfected with flagellin or poly(dA:dT) to activate NLRC4 and AIM2 inflammasomes, respectively. The activation of these inflammasomes and pyroptosis was revealed by PI incorporation and observed by fluorescence microscopy. The results showed that neither NLRC4 nor AIM2 inflammasome-mediated pyroptosis had been affected by TAS pre-treatment (Supplementary Figure 1). Together, these results suggested that TAS, to certain extent, specifically inhibited NLRP3 inflammasome activation and pyroptosis in murine macrophages.

### TAS Suppresses LPS-Induced Over-Activation of mTOR Signaling

**Figure 4:**
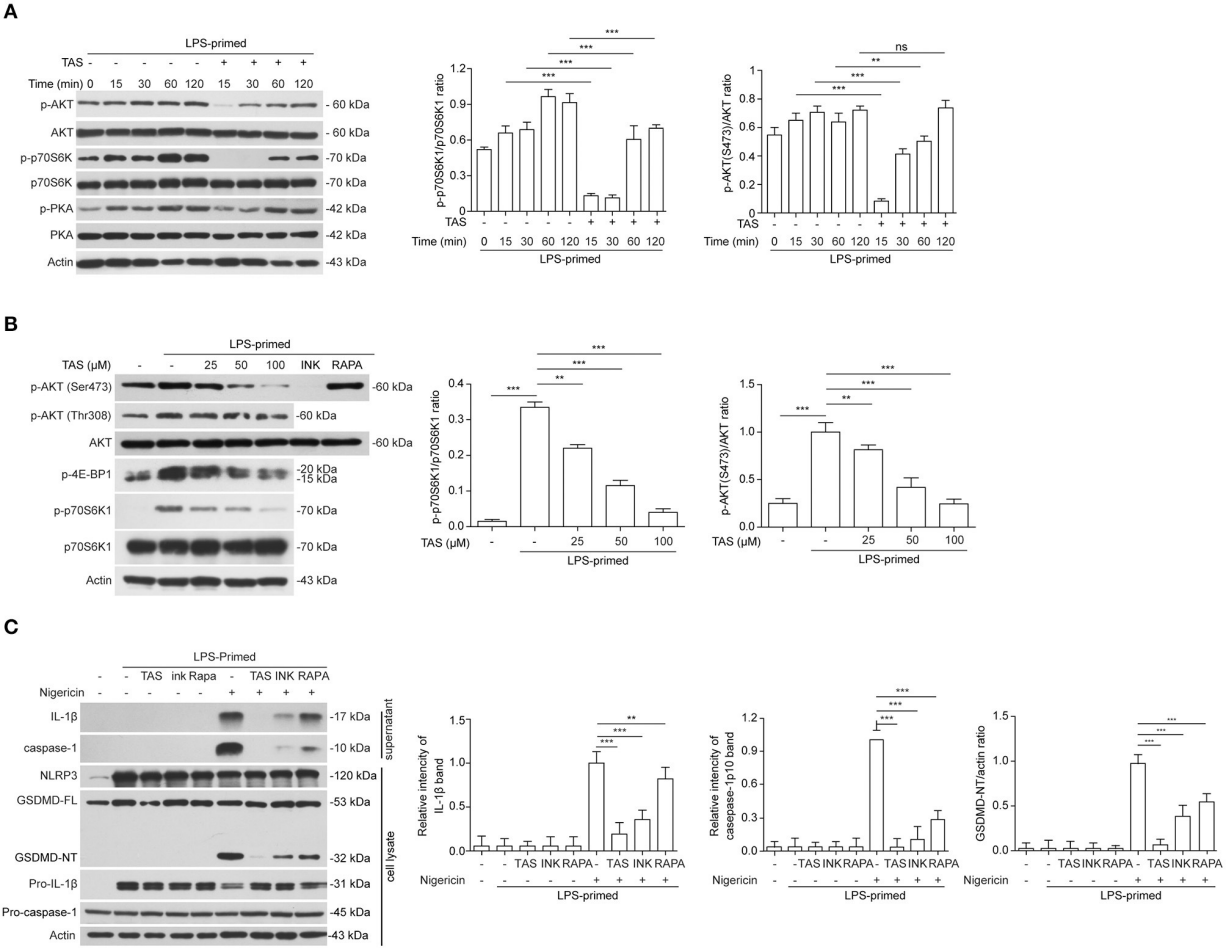
TAS inhibited mTOR signaling in LPS-primed macrophages. LPS-primed BMDMs were primed with 0.5 μg/ml LPS for 4h, followed by treatment with 100 μM TAS for indicated time lengths **(A)**, or indicated concentration of TAS for 15 min **(B)**, or 100 μM TAS, 20 nM rapamycin (RAPA), 30 nM INK-128 (INK), respectively **(C)**. The levels of indicated proteins in the cell lysates were analyzed by Western blotting. Actin was added as loading control. Data are shown as mean ± SD (*n* = 5). **P < 0.01; ***P < 0.001; ns, not significant.

It has been reported that several signaling pathways including PKA, JNK, and mTOR pathways can regulate NLRP3 inflammasome activation (19). Thus, we next sought to explore whether these pathways were involved in TAS-mediated suppression on NLRP3 inflammasome activation in macrophages. By Western blotting analysis, we found that LPS robustly induced the activation of both mTOR complex 1 (mTORC1) (as revealed by p70S6K1 phosphorylation) and mTORC2 (indicated by Akt-Ser473 phosphorylation), but TAS treatment time-dependently inhibited the mTOR signaling (Figure 4A). Further Western blotting showed that a short time treatment (15 min) with TAS in LPS-primed macrophages dose-dependently suppressed both mTORC1 (as indicated by p-p70S6K1 and p-4E-BP1) and mTORC2 activation but not phosphoinositide 3-kinase (PI3K) activity (revealed by Akt-Thr308 phosphorylation) (Figure 4B). Besides, it seemed that PKA and JNK signaling pathways, which have been reported to regulate NLRP3 inflammasome pathways (see section Introduction), were not obviously influenced by TAS treatment (Figure 4A, Supplemental Figure 2). Interestingly, although TAS, INK-128 and rapamycin showed different mTOR-inhibitory effects and features, they all significantly suppressed NLRP3 inflammasome activation, albeit at different extents, as indicated by the levels of released IL-1β and caspase-1p10 in the supernatants and GSDMD-NT in the cell lysates (Figure 4C). Together, these results suggest that TAS suppressed NLRP3 inflammasome activation via regulating mTOR signaling.

### TAS Alleviates Mitochondrial Damage Upon NLRP3 Inflammasome Stimulation

**Figure 5:**
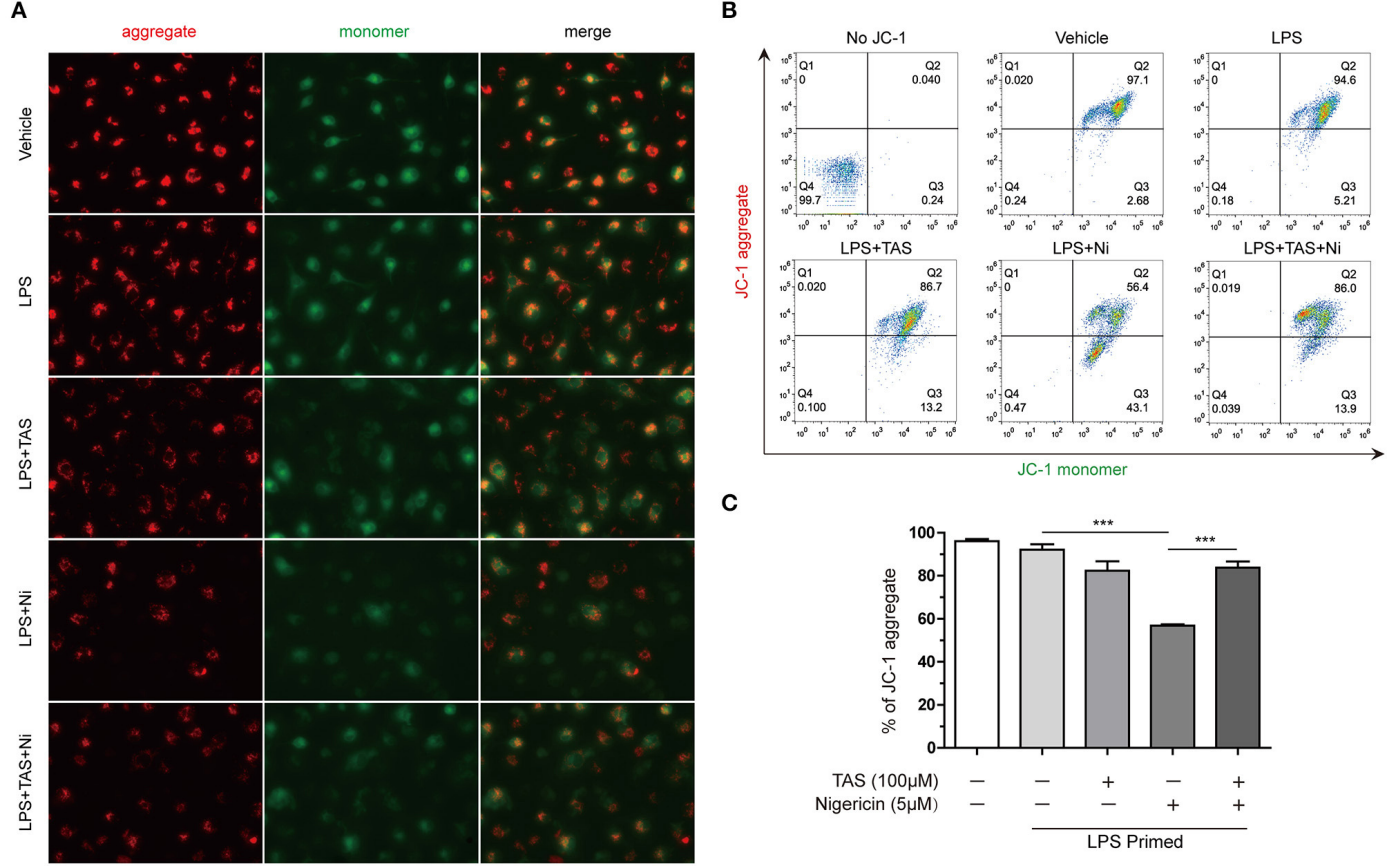
Taraxasterol alleviates mitochondrial damage induced by nigericin. Mouse BMDMs were first primed with 0.5 μg/ml LPS for 4 h, then treated with 100 μM taraxasterol (TAS) for 1 h, and finally stimulated with 5 μM nigericin for 1 h. Mitochondrial membrane potential was assayed with a JC-1 kit according to the instruction of the manufacturer. JC-1 aggregates and monomers were observed by fluorescent microscopy **(A)** and evaluated by flow cytometry **(B)**; the percentages of cells with JC-1 aggregates are shown **(C)**. Data are shown as mean ± SD (*n* = 3). ***P < 0.001.

Previous studies have shown that during NLRP3 inflammasome activation, LPS induced a sharp change of metabolism, while NLRP3 inflammasome activators, such as nigericin, induced mitochondrial damage (41). Increasing evidence has shown that energy regulatory drugs, such as rapamycin and metformin, are able to either reduce or enhance NLRP3 inflammasome activation (21, 45). On the other hand, increase of mitophagy thus accelerating removal of the damaged mitochondria by inhibition of mTOR can reduce NLRP3 inflammasome activation (46, 47). As TAS was able to inhibit mTOR signaling (Figure 4), we next investigated whether TAS influenced nigericin-induced mitochondrial damage. The mitochondrial membrane potential was detected with a JC-1 kit. The cells were observed by fluorescent microscopy and analyzed by flow cytometry as well. The results showed that nigericin reduced mitochondrial membrane potential as evidenced by decreased JC-1 aggregates (red), while TAS significantly prevented the reduction of mitochondrial membrane potential indicating alleviation of the mitochondrial damage induced by nigericin (Figures 5A–C). These results suggested that TAS suppressed NLRP3 inflammasome activation by alleviating mitochondrial damage.

### TAS Improves Mouse Survival From Acute Bacterial Infection

**Figure 6:**
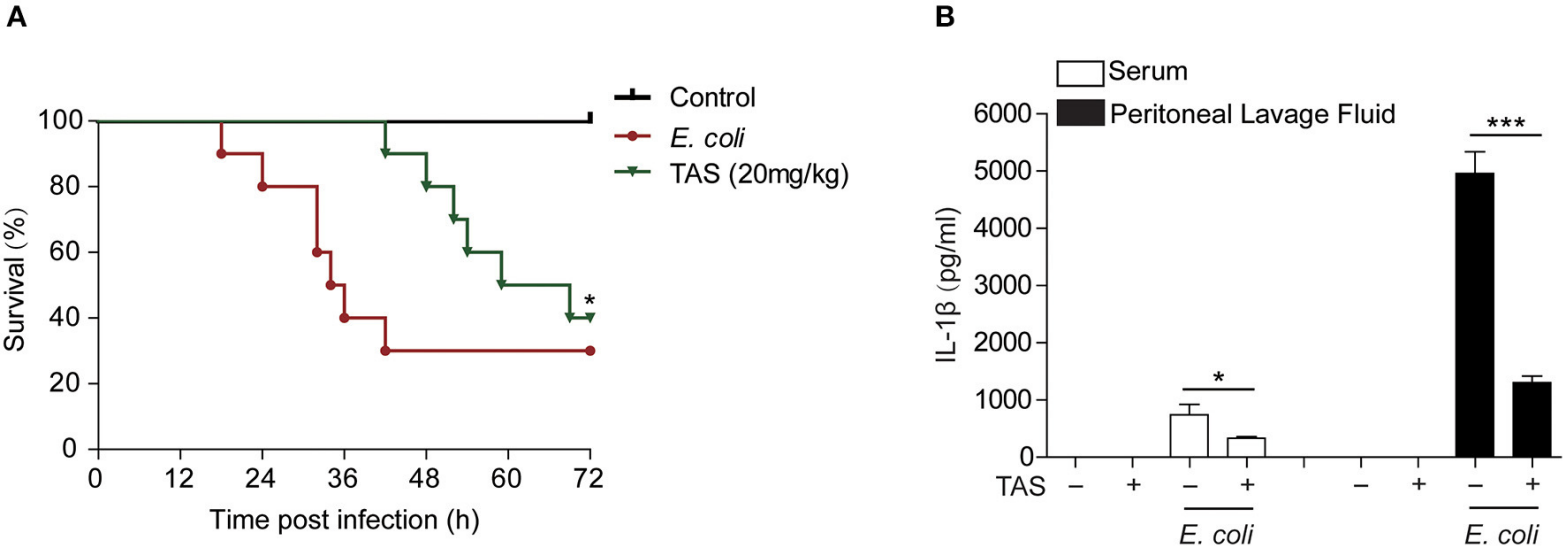
Taraxasterol increases mouse survival from bacterial infection. Female C57BL/6 mice were administered intragastrically (i.g.) with TAS or vehicle (2% Tween-80 in PBS); 3 h later, the mice were injected with freshly prepared viable *E. coli* (2 × 10^9^ CFU/mouse in 0.3 ml PBS) into the peritoneal cavity of each mouse; One hour after bacterial injection, the mice were given with a same dose of TAS again (i.g.). **(A)** Mouse survival was observed and recorded every 6 h for 3 consecutive days (*n* = 10). *P < 0.05, TAS (20 mg/kg) group vs. *E. coli* group. **(B)** In a parallel experiment, mouse serum and peritoneal lavage fluid was collected at 8 h post bacterial infection. IL-1β in the serum and peritoneal lavage fluid, respectively, were evaluated by CBA assay (*n* = 5).*P < 0.05; ***P < 0.001.

Considering that ATP-induced NLRP3 inflammasome activation and IL-1β release play critical roles in sepsis of bacterial infection (37) and that TAS suppressed NLRP3 inflammasome activation by ATP or nigericin, we lastly investigated whether TAS was able to suppress IL-1β release *in vivo* and protected mice from acute bacterial infection. Viable *E. coli* cells was injected into the peritoneal cavity of mice and TAS was administered intragastrically (*i.g*.) twice before and after bacterial infection. The results showed that TAS at a dose of 20 mg/kg body weight significantly improved mouse survival from bacterial infection as compared to *E. coli* group (Figure 6A). After bacterial infection, IL-1β levels (detected at 8 h post infection) were sharply increased in the sera and peritoneal lavage fluids of the mice, but TAS treatment significantly reduced the IL-1β levels (Figure 6B). These results suggested that TAS was able to inhibit NLRP3 inflammasome activation *in vivo* and thus conferring protection against bacterial infections induced by bacterial infection.

## Discussion

TAS is an active ingredient of Taraxacum plants, which has long been used as a Chinese herbal medicine for treatment of inflammatory diseases such as mastitis (48). Various anti-inflammatory activities of TAS have been reported (see section Introduction), but the underlying mechanism is unclear. Here we showed that TAS significantly suppressed NLRP3 inflammasome activation and reduced the release of IL-1β and pyroptosis, thus revealing a novel action mechanism for TAS in treating inflammatory diseases in view of the critical role of NLRP3 inflammasome activation in such morbidities.

Upon its activation, NLRP3 protein recruits the adaptor protein ASC, which further recruits pro-caspase-1 to form a large protein complex called NLRP3 inflammasome. This constitutes a platform for pro-caspase-1 auto-cleavage and activation. Active caspase-1 then cleaves pro-IL-1β and the mature product is released from the cell to enhance inflammatory responses (49, 50). As dysregulation of NLRP3 inflammasome activation is associated with various inflammatory disorders (51, 52), suppression of NLRP3 inflammasome activation is therefore a potential avenue to tackling such disorders. By using canonical NLRP3 activators including nigericin and ATP to activate this inflammasome in mouse macrophages, we showed that TAS was able to inhibit the NLRP3 inflammasome markedly as revealed by the reduced release of cleaved caspase-1 p10 fragment and mature IL-1β (17 kDa), two hallmarks of inflammasome activation. Concomitantly, TAS pre-treatment also significantly attenuated the pyroptotic cell death, which was consistent with the decreased generation of GSDMD-NT from GSDMD by active caspase-1-mediated cleavage. However, TAS had no significant effects on NLRC4 (induced by flagellin) or AIM2 [activated by poly(dA:dT)] inflammasome-mediated pyroptosis. Thus, TAS was a relatively specific inhibitor of NLRP3 inflammasome activation.

As a hallmark of NLRP3 inflammasome activation, a large ASC speck is usually formed in the macrophage upon stimulation with nigericin or ATP. It is known that ASC contains both PYD and CARD domains, acting as an adaptor protein for the interaction of NLRP3 and pro-caspase-1 that have a PYD and CARD domain, respectively. Although the mechanism of ASC speck formation is still quite unclear, it is generally regarded that NLRP3 recruits ASC through their PYD-PYD interaction, which becomes a nuclear core for recruiting more ASC proteins through their CARD-CARD or PYD-PYD interaction (53, 54). As mentioned above, ASC speck is a platform for pro-caspase-1 binding and auto-catalytic activation (55, 56). Phosphorylation of ASC Tyr144 residue in the CARD domain, as catalyzed by Syk or JNK, is critical for the formation of ASC speck (57). Besides, the PKA signaling pathway has been shown to negatively regulate NLRP3 activation by specifically phosphorylating the protein NLRP3 (6, 7, 58). We found in this study that TAS markedly inhibited ASC speck formation and dose-dependently reduced the oligomerization of ASC in murine primary macrophages. However, it seemed that TAS did not significantly affect JNK and PKA signaling (Figure 4A, Supplementary Figure 2), suggesting that these signaling pathways may not be involved in the drug's action in our experimental setting.

Previous studies have indicated that NLRP3 inflammasome activators lead to vigorous changes of immunometabolism while mTOR inhibitors such as rapamycin and Torin 1 can inhibit the inflammasome activation driven by mTORC1 (20). Mitochondrion is the organelle responsible for energy metabolism via tricarboxylic acid (TCA) cycle and oxidative phosphorylation (59). LPS induces a sharp change of glucose intake and glycolysis, while NLRP3 inflammasome activators, such as nigericin, induce mitochondrial damage, which induces mtDNA damage and releases reactive oxygen species (ROS), calcium and potassium ions to trigger the inflammasome activation (60, 61). Interestingly, active caspase-1 can in turn cleave glycolysis-related enzymes including aldolase, glyceraldehyde-3-phosphate dehydrogenase (GAPDH) and α-enolase (62). NEK7, a serine-threonine kinase that has previously been associated with mitosis, is identified to be an NLRP3 inflammasome component that can sense the mitochondrial ROS; the binding of NEK7 to NLRP3 depends on ROS, while deficiency of NEK7 in mice prevents the assembly of NLRP3 inflammasome (63). Increase of mitophagy by inhibition of mTOR signaling may reduce NLRP3 inflammasome activation (46, 47). Consistent with these studies, we found that LPS robustly induced the activation of mTORC1 and mTORC2, but TAS time- and dose-dependently inhibited their activation by LPS priming; moreover, TAS significant alleviated the mitochondrial damage induced by nigericin, as revealed by mitochondrial membrane potential assayed with JC-1, suggesting that TAS might have inhibited NLRP3 inflammasome activation by protecting mitochondrial damage through regulating the mTOR signaling.

Although mTOR may regulate NLRP3 inflammasome through the mitophagy pathway, it is still not completely understood whether mTOR signaling regulates NLRP3 activation through other apthways. Previous studies showed that the activation of mTORC1 can promote the expression and maturation of IL-1β through hypoxia-inducible factor-1α (HIF-1α), whereas rapamycin can inhibit NF-κB signaling through specifically targeting mTORC1 (64), suggesting that mTORC1 can regulates the expression of NLRP3 inflammasome components such as IL-1β and NLRP3. A separate study revealed that incubation of rapamycin at the LPS-priming step can reduce pro-IL-1β mRNA expression and eliminate mitochondrial reactive oxygen species (mtROS), thus suppressing ATP-induced NLRP3 inflammasome activation (65). Besides, inhibition of mTORC1 by rapamycin induces autophagy, which targets pro-IL-1β for degradation (65, 66). However, in this study, TAS was added after LPS-priming and it did not significantly change the expression of LPS-induced pro-IL-1β and NLRP3 or constitutively-expressed ASC and GSDMD, suggesting that TAS might regulate the inflammasome activation at the second step (triggering). Another study indicated that NLRP3 is a direct binding partner of mTOR (67), but whether TAS reduced the expression of mTOR protein (NLRP3 protein seemed less affected by TAS) or inhibited the binding of mTOR and NLRP3 is unclear. Although previous studies have shown that rapamycin (a specific inhibitor of mTORC1) regulates NLRP3 inflammasome activation (20), indicating the role of mTORC1 in this process, it is unclear whether mTORC2 also regulates NLRP3 inflammasome activation and how it does so. Recent studies have pointed out that mTORC2 activation promotes Na^+^ influx and K^+^ efflux by catalyzing the phosphorylation of serum- and glucocorticoid-inducible kinase 1 (SGK1, belonging to the same kinase family with Akt) in renal tubule cells (68, 69). As TAS was able to inhibit mTORC2 signaling, it is proposed that this drug may have suppressed NLRP3 inflammasome activation by regulating K^+^ efflux partly through the mTORC2 pathway. Further investigation is therefore warranted to uncover the precise action mechanism underlying TAS's effects on NLRP3 activation.

As an active ingredient of Dandelion, TAS's effects on NLRP3 inflammasome activation may to some extent reflect the pharmacological activities of this medicinal plant. Thus, our findings may at least partly explain the therapeutic effects of this plant on inflammatory diseases. These diseases have been shown to be caused by either bacterial infections or aseptic inflammation, suggesting the potential activation of NLRP3 inflammasome. Along this line, other NLRP3-related disorders may gain benefits from the therapeutic use of TAS which awaits future exploration.

In sum, we demonstrated that TAS suppressed NLRP3 inflammasome likely by inhibiting LPS-induced mTOR activation. Our findings, together with previous studies (20, 21), still suggest that targeting mTOR signaling may be an avenue for controlling diseases related to dysregulation of NLRP3 inflammasome activation and that TAS may be a potential anti-inflammation drug for inflammatory diseases with dysregulated NLRP3 activation.

## Data Availability Statement

The original contributions generated for the study are included in the article/Supplementary Material, further inquiries can be directed to the corresponding author/s.

## Ethics Statement

The animal study was reviewed and approved by the Committee on the Ethics of Animal Experiments of Jinan University.

## Author Contributions

FY, X-jY, M-yC, Y-fW, M-yZ, C-sZ, BZ, and L-hX performed the experiments. FY, H-cL, X-hH, and D-yO analyzed the data. FY, H-cL, X-hH, and D-yO wrote the manuscript. X-hH and D-yO conceived and supervised the project. All authors contributed to the article and approved the submitted version.

## Conflict of Interest

The authors declare that the research was conducted in the absence of any commercial or financial relationships that could be construed as a potential conflict of interest.
