# Supplementary material for: Inhibition of NLRP3 Inflammasome Activation and Pyroptosis in Macrophages by Taraxasterol Is Associated With Its Regulation on mTOR Signaling
Source: Front Immunol. 2021 Feb 17;12:632606. doi: 10.3389/fimmu.2021.632606 (PMC7925414; doi:10.3389/fimmu.2021.632606)
Supplement: Supplementary file 1 [file Data_Sheet_1.PDF]

Supplemental Material

Inhibition of NLRP3 Inflammasome Activation and Pyroptosis in Macrophages by Taraxasterol Is Associated with Its Regulation on mTOR Signaling

Fan Yang[1,#], Xun-jia Ye[1,#], Ming-ye Chen[1,#], Hong-chun Li[2], Yao-feng Wang[1], Mei-Yan Zhong[1], Chun-su Zhong[1], Bo Zeng[1], Li-hui Xu[3], Xian-hui He[1,*], Dong-yun Ouyang [1,*]

[1]Department of Immunobiology, College of Life Science and Technology, Jinan University, Guangzhou, China, 510632

[2]Wuzhongpei Memorial Hospital of Shunde, Foshan 528300, China

[3]Department of Cell Biology, College of Life Science and Technology, Jinan University, Guangzhou, China, 510632

* Correspondence:
Xian-hui He
thexh@jnu.edu.cn
Dong-yun Ouyang
dongyun1967@aliyun.com

Running title: Taraxasterol inhibits NLRP3 inflammasome activation

Keywords: Taraxasterol; Dandelion; NLRP3 inflammasome; ASC speck; mTOR

Supplemental Fig. S1

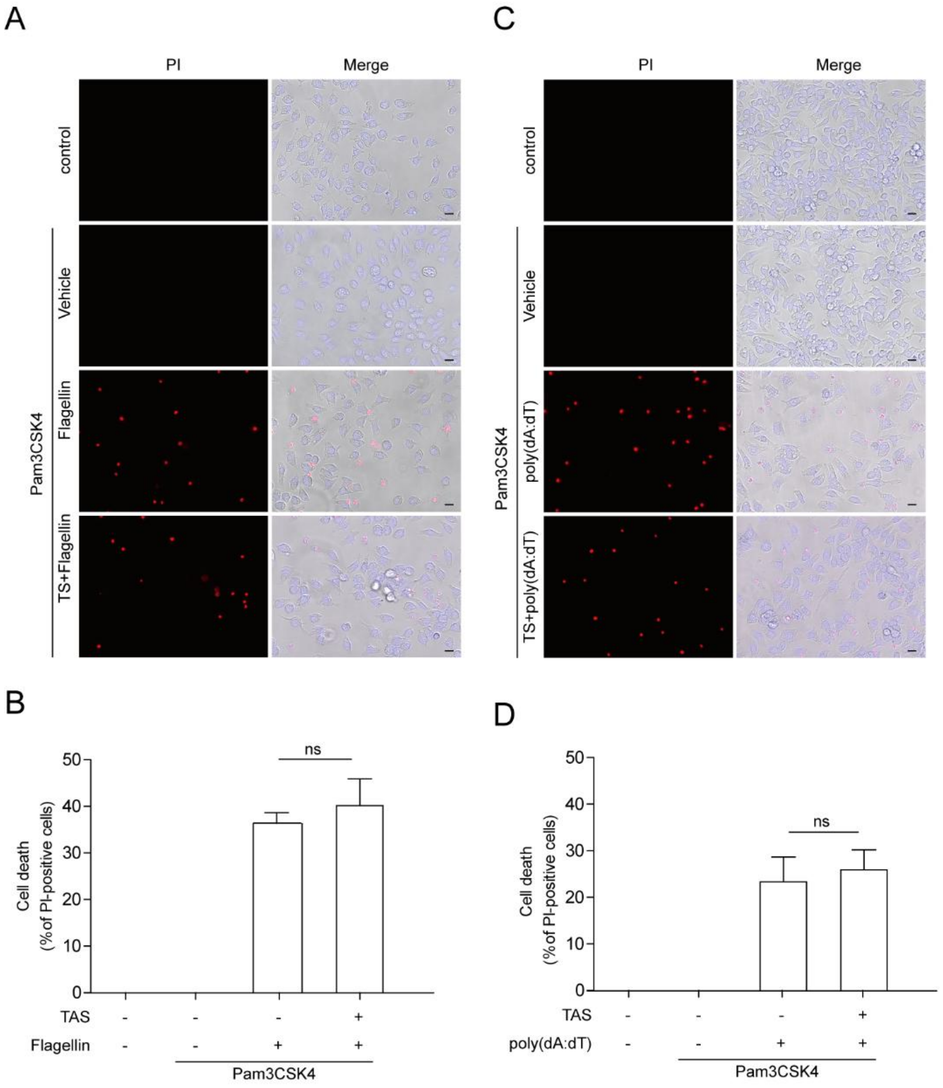

Fig. S1. Taraxasterol (TAS) did not influence the activation of NLRC4 and AIM2 inflammasomes in J774A.1 macrophages. J774A.1 macrophages were primed by Pam3CSK4 (1 μg/mL) for 4 h and then pre-treated with TAS (100 μM) for 1 h, followed by transfection with flagellin (0.5 μg/mL) (**A, B**) or poly (dA:dT) (2 μg/mL) (**C, D**) for 16 h. (**A, C**) The cells were stained with 2 μg/ml propidium iodide (PI) (red; staining dead cells) plus 5 μg/ml Hoechst 33342 (blue; staining all cells) for 10 min, then observed by fluorescent microscopy. (**B, D**) The percentage of cell death is defined as PI-positive cells in 5 random fields to all cells (Hoechst 33342-positive). Data are shown as mean ± SD (*n* = 5). ns, not significant.

Supplemental Fig. S2

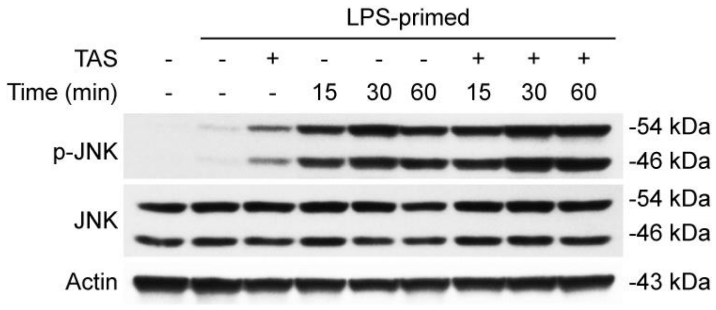

Fig. S2. TAS did not significantly affect JNK signaling. J774A.1 macrophages were primed by LPS (0.5 μg/ml) and then pre-treated with TAS (100 μM) for 1 h followed by treatment with nigericin 5μM for indicated time. The levels of p-JNK and JNK in the cell lysates were analyzed by Western blotting. Actin was added as loading control.
